# Using an internet consumer marketing strategy to reach men who have sex with men for participation in a preventive HIV vaccine clinical trial

**DOI:** 10.1186/1742-4690-9-S2-P119

**Published:** 2012-09-13

**Authors:** CD Voytek, KT Jones, BL Curtis, DT Fiore, D Dunbar, I Frank, DS Metzger

**Affiliations:** 1 University of Pennsylvania, Philadelphia, PA, USA; 2 University of Pennsylvania Annenberg Public Policy Center, Philadelphia, PA, USA; 3 University of Pennsylvania Perelman School of Medicine, Philadelphia, PA, USA

## Background

Sustaining research subject recruitment in biomedical HIV prevention trials requires continual innovation. Since June 2009, the University of Pennsylvania HIV Vaccine Trials Unit (UPenn HVTU) has employed multiple strategies to recruit men who have sex with men (MSM) for a phase IIb HIV vaccine trial, HVTN 505.

## Methods

Between 12/1/2011 and 1/3/2012, the HVTU contracted with a consumer marketing company to recruit potential trial participants. For $3000, the company emailed MSM a scripted message inviting them to participate in a clinical HIV vaccine trial, and the company provided the trial site with contact information for those who responded. Site staff emailed and phoned each respondent to provide study information and conduct a phone-screen interview for the trial. Those eligible were scheduled for in-office screening appointments.

## Results

266 MSM were emailed; 118 viewed the message, and 109 responded that they were interested in participating. 83% of responders were White, and 71% earned >$50,000/year. Staff successfully contacted 64 individuals, and 58 completed phone-screens. Of these, 17 were eligible for, and 9 completed, screening visits. Five enrolled in HVTN 505 during January-February, 2012. Of 41 phone-screened ineligible, primary reasons for ineligibility were: being HIV-positive (n=20; 49%), not meeting protocol-specified sexual risk criteria (n=11; 27%), out of age range (n=6; 15%), and/or being uncircumcised (n=5; 12%). Ineligible participants were referred to phase I or future prevention trials as appropriate.

## Conclusion

This strategy reached MSM not engaged by previous efforts, and doubled site HVTN 505 enrollment over two months. Respondents included a higher proportion of White MSM than the population screened for HVTN 505 at the HVTU. This approach has wider potential use for recruitment in biomedical HIV prevention trials. To understand the true utility of this approach, respondent HIV risk data and financial costs associated with this strategy must be carefully examined.

